# William Fitzwilliam Carter

**Published:** 1900-06

**Authors:** 


William Fitzwilliam Carter died at his residence in Coronation Road on April 26th, from typhoid fever, in his sixtieth year. Mr. Carter received his medical education at the school of the Royal College of Surgeons in Ireland, and became qualified as L.R.C.P. Irel.in 1863; two years later he was admitted L.R.C.P. and L.M. Edin. Mr. Carter was for a short time in practice in Brisbane, Australia, where he was honorary physician to the Lady Bowen's Hospital, and on his return to England he settled in Bristol. He was formerly district medical officer and public vaccinator for Bedminster, and was only quite recently granted a superannuation allowance by the Board of Guardians. He was also formerly medical officer of the Bedminster Fever Hospital.

